# Supplementary material for: A Tight-Connection g-C3N4/BiOBr (001) S-Scheme Heterojunction Photocatalyst for Boosting Photocatalytic Degradation of Organic Pollutants
Source: Nanomaterials (Basel). 2024 Jun 22;14(13):1071. doi: 10.3390/nano14131071 (PMC11243395; doi:10.3390/nano14131071)
Supplement: Supplementary file 1 [file nanomaterials-14-01071-s001.zip › nanomaterials-3041804-supplementary.pdf]

A tight connection g-C_3N_4/BiOBr (001) S-scheme heterojunction photocatalysts for boosting photocatalytic degradation of organic pollutants

Xinyi Zhang [a], Weixia Li [a], Liangqing Hu [a], Mingming Gao [b*], Jing Feng [a*]

[a] *Key Laboratory of Superlight Materials & Surface Technology of Ministry of Education, Harbin Engineering University, Harbin 150001, PR China*

[b] *College of Biological and Chemical Engineering, Qilu Institute of Technology, Jinan 250200, China*

* Corresponding author. gaomingm@qlit.edu.cn (MingMing Gao), fengjing@hrbeu.edu.cn (Jing Feng)

1.

Table S1. ICP of x-g-C_3N_4/BiOBr (x=4, 6, 8)

Sample	Concentration of Bi^{3+} (mg L^{-1})	BiOBr: g-C_3N_4
**4-g-C_3N_4/BiOBr**	30.47	10:3.7
**6-g-C_3N_4/BiOBr**	25.12	10:5.3
**8-g-C_3N_4/BiOBr**	20.61	10:7.5

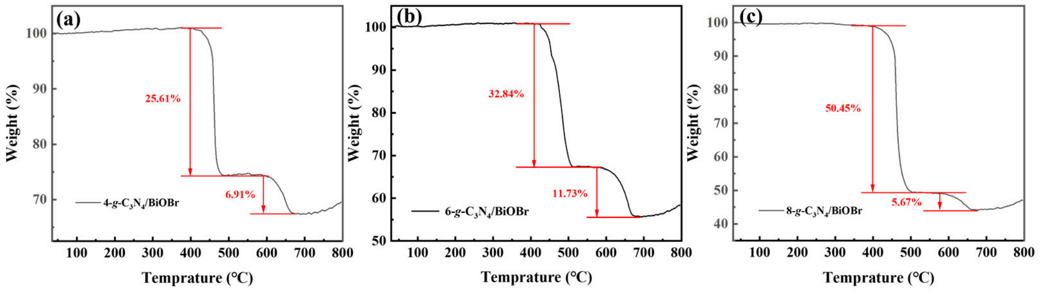

Figure S1. TG curve of (a) 4-*g*-C_3N_4/BiOBr, (b) 6-*g*-C_3N_4/BiOBr (*g*-C_3N_4/BiOBr), and (c) 8-*g*-C_3N_4/BiOBr.

Table S2. Composition of x-*g*-C_3N_4/BiOBr (x=4, 6, 8) according TG

Sample	BiOBr:g-C_3N_4 (theoretical)	BiOBr:g-C_3N_4 (actual)
**4-g-C_3N_4/BiOBr**	10:4	10:3.5
**6-g-C_3N_4/BiOBr**	10:6	10:4.8
**8-g-C_3N_4/BiOBr**	10:8	10:7.4

Table S3. The degradation efficiency of high concentration TC by different catalysts

Catalysts	Concentration of TC (mg L^{-1})	Catalyst dosage (mg L^{-1})	Degradation efficiency	Solution volume (L)	Time (min)	References
Cu_2O/BiOCl	20	20	0.903	0.1	80	[1]
$BiVO_4$@BiOCl	40	50	0.9032	0.05	180	[2]
CDs-$ZnIn_2S_4$	50	100	0.83	0.1	120	[3]
Mo_2C/MoO_x films	50	2cm × 2cm	0.917	0.02	60	[4]
Fe-based MOFs (MIL-88A)	200	250	0.6	0.1	90	[5]
g-C_3N_4/BiOBr	50	50	0.89	0.1	120	This work

[1] Yuan, X.X.; Yang J.Y.; Yao, Y.Y.; et al. Preparation, characterization and photodegradation mechanism of 0D/2D Cu_2O/BiOCl S-scheme heterojunction for efficient photodegradation of tetracycline. SEP PURIF TECHNOL, **2022**, 291: 120965.

[2] Yang, Q.; Tan, G.Q.; Yin, L.X.; et al. Full-spectrum broad-spectrum degradation of antibiotics by $BiVO_4$@BiOCl crystal plane S-type and Z-type heterojunctions. Chem. Eng. J., **2023**, 467: 143405.

[3] Shi, W.L.; Hao, C.C.; Fu, Y.M.; et al. Enhancement of synergistic effect photocatalytic/persulfate activation for degradation of antibiotics by the combination of photo-induced electrons and carbon dots. Chem. Eng. J., **2022**, 433: 133741.

[4] Lian, Z.; Wu, T.; Zhang, X.N.; et al. Synergistic degradation of tetracycline from Mo_2C/MoO_x films mediated peroxymonosulfate activation and visible-light triggered photocatalysis. Chem. Eng. J., **2023**, 469: 143774.

[5] Zhang, Y.; Zhou, J.B.; Chen, X.; et al. Coupling of heterogeneous advanced oxidation processes and photocatalysis in efficient degradation of tetracycline hydrochloride by Fe-based MOFs: Synergistic effect and degradation pathway. Chem. Eng. J., **2019**, 369: 745-757.

Table S4. The water intake location

Sample	Intake Location
**Tap Water**	Harbin Engineering University (45°46＇19＂N, 126°40＇55＂E)
**Lake Water**	Majiagou (45°46＇23＂N, 126°41＇2＂E)
**River Water**	Songhua River (45°46＇40＂N, 126°36＇23＂E)

Table S5. Natural water matrix parameters

Parameter	pH Value	Electrical conductivity (μS cm^{-1})	COD (mg L^{-1})
**Deionized Water**	9.23	3.07	–
**Tap Water**	7.04	164.5	–
**River Water**	7.17	293	3
**Lake Water**	6.92	768	0.05

2. Formulas

The position of the valence band (E_{VB}) and the conduction band (E_{CB}) are obtained by the following Formula S1 and S2:

$$E_{VB} = X - E_e + \frac{1}{2}E_g \quad \text{(S1)}$$

$$E_{CB} = E_{VB} - E_g \quad \text{(S2)}$$

where E_g is the band gap, the value of E_e (Normal hydrogen electrode) is 4.5 eV, and the X (Electronegativity) of BiOBr and g-C_3N_4 are 6.17 eV and 4.77 eV,

respectively.

3. Supplementary Figures

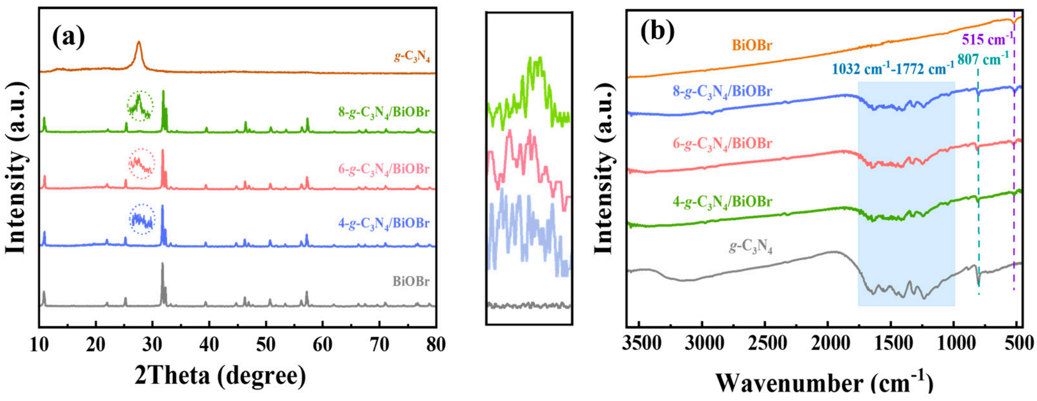

Figure S2. (a) XRD patterns and (b) FT-IR spectra of g-C_3N_4, BiOBr, x-g-C_3N_4/BiOBr (x= 4, 6, 8).

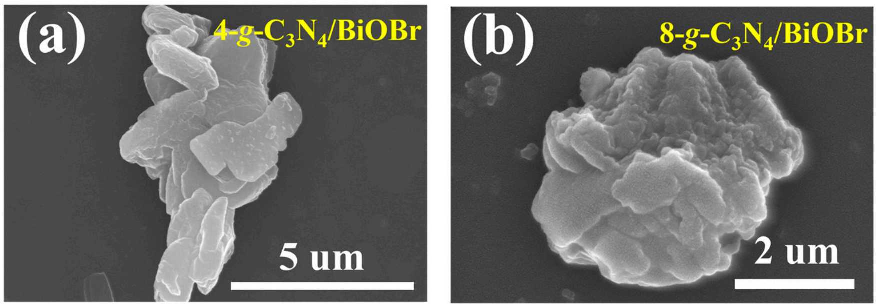

Figure S3. SEM images of (a) 4-g-C_3N_4/BiOBr and (b) 8-g-C_3N_4/BiOBr.

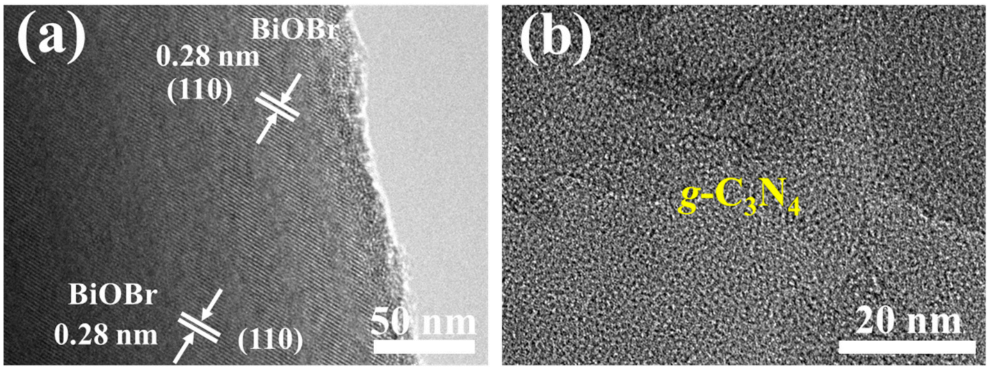

Figure S4. HRTEM images of (a) BiOBr and (b) g-C_3N_4.

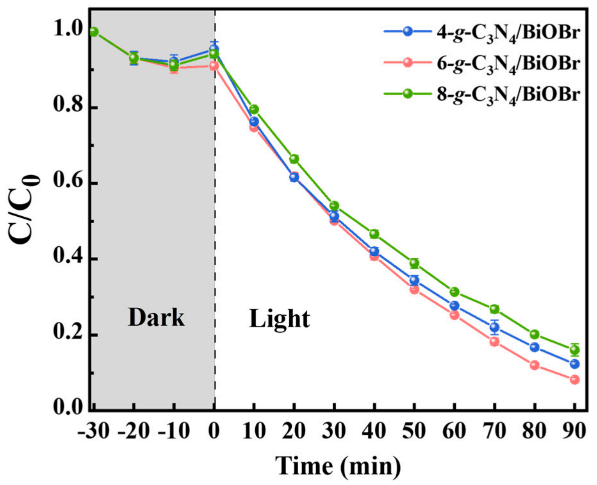

Figure S5. Photocatalytic degradation of MB by 4-*g*-C_3N_4/BiOBr, 6-*g*-C_3N_4/BiOBr, and 8-*g*-C_3N_4/BiOBr.

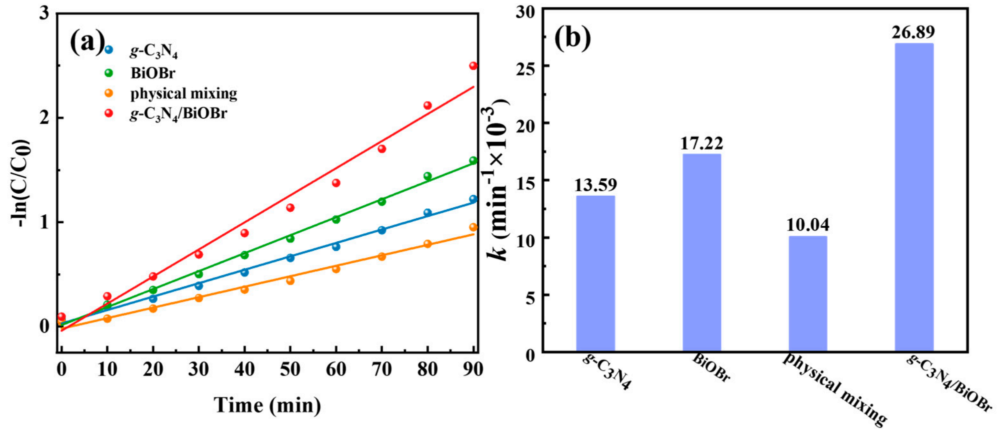

Figure S6. The pseudo-first-order kinetic rate plots (a), and the corresponding rate constants in the photocatalytic degradation system (b) of *g*-C_3N_4, BiOBr, 6-*g*-C_3N_4/BiOBr, and physical mixing.

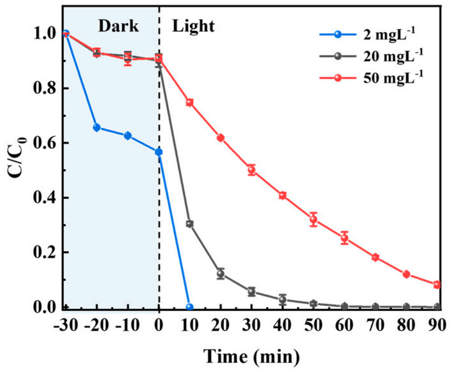

Figure S7. The degradation efficiency of TC at different concentrations

(2 mgL^{-1},20 mgL^{-1}, and 50 mgL^{-1})

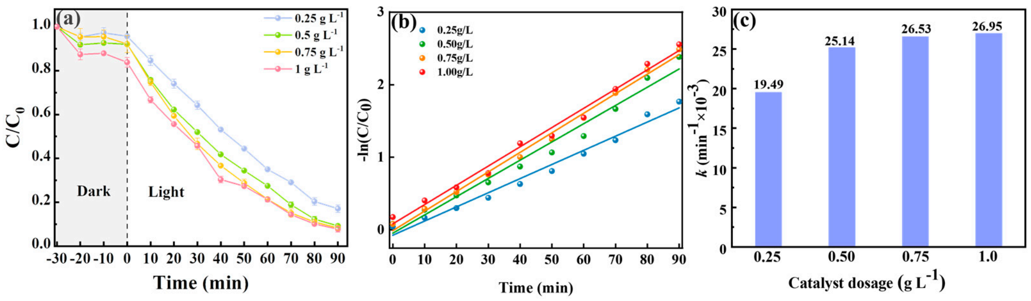

Figure S8. (a) Effect of catalyst dosage (0.25–1.00 g L^{-1}), (b) the pseudo-first-order kinetic rate plots, and (c) the corresponding rate constants in the photocatalytic degradation system of different 6-*g*-C_3N_4/BiOBr dosage.

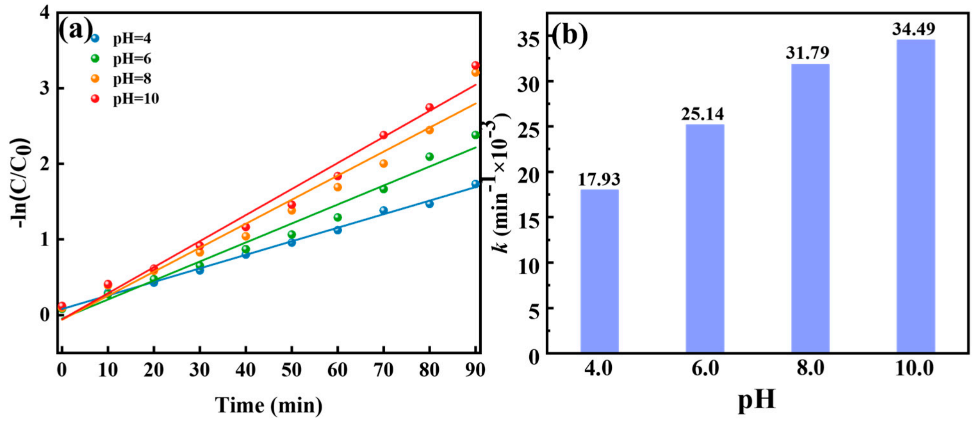

Figure S9. (a) The pseudo-first-order kinetic rate plots, and (b) the corresponding rate constants in the photocatalytic degradation system of 6-*g*-C_3N_4/BiOBr under different initial pH (4-10).

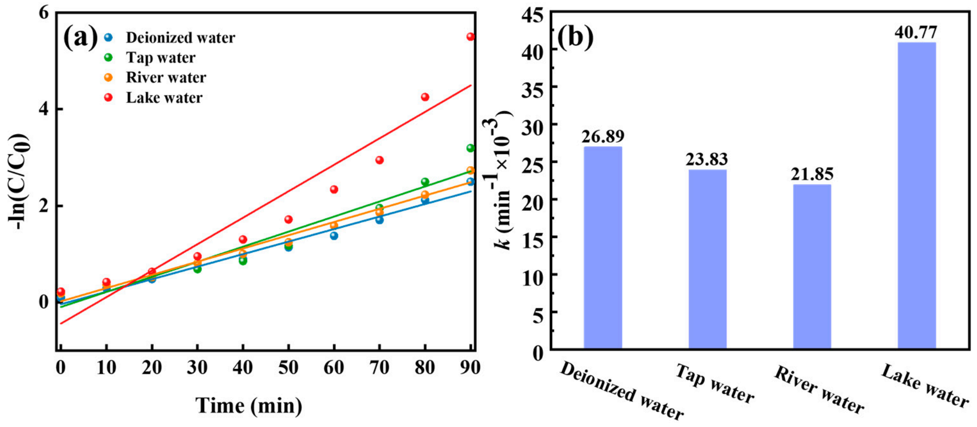

Figure S10. The pseudo-first-order kinetic rate plots (a), and the corresponding rate constants in the photocatalytic degradation system (b) of 6-*g*-C_3N_4/BiOBr at actual water quality (tap, lake, and river water).

We found that the photocatalytic degradation behavior in lake water is nonlinear. This may be due to the enhanced separation of photogenerated charge carriers in the presence of different anions in water, especially HCO_3^- in lake and river water and Cl^- in tap water. The reaction between various anions (such as Cl^-, HCO_3^-, NO_3^-) and photocatalysts can generate more free radicals (such as $Cl^{\cdot}$, $HCO_3^{\cdot}$, $CO_3^{\cdot}$, $NO_3^{\cdot}$) [6]. Meanwhile, as a typical compound containing nitrogen and sulfur, MB can exhibit high reactivity with Cl and be effectively oxidized and decomposed through electron transfer or hydrogen extraction [7, 8].

[6] Lu, W.Y.; Xu, L.J.; Shen, X.B.; et al. Highly efficient activation of sulfite by p-type S-doped g-C_3N_4 under visible light for emerging contaminants degradation. Chem. Eng. J., **2023**, 472: 144708.

[7] Wang, S.L.; Wu, J.F.; Lu, X.Q.; et al. Removal of acetaminophen in the Fe^{2+}/persulfate system: Kinetic model and degradation pathways. Chem. Eng.

J., **2019**, 358: 1091-1100.

[8] Luo, C.W.; Wang, S.S.; Wu, D.J.; et al. UV/Nitrate photocatalysis for degradation of Methylene blue in wastewater: Kinetics, transformation products, and toxicity assessment. ENVIRON TECHNOL INNO, **2022**, 25: 102198.

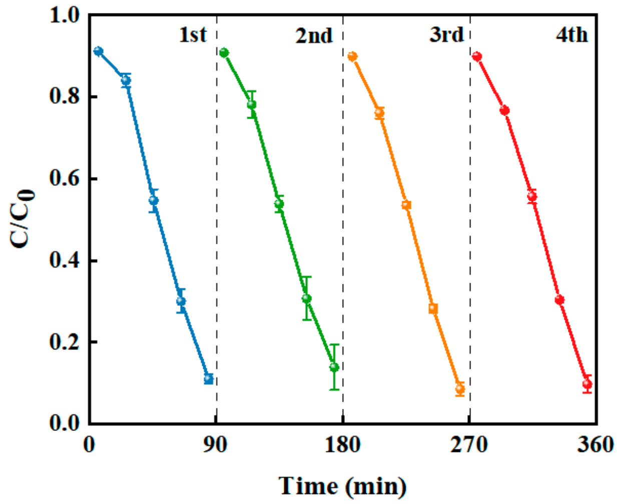

Figure S11. Cycle performance with photocatalytic degradation of MB by 6-*g*-C_3N_4/BiOBr.

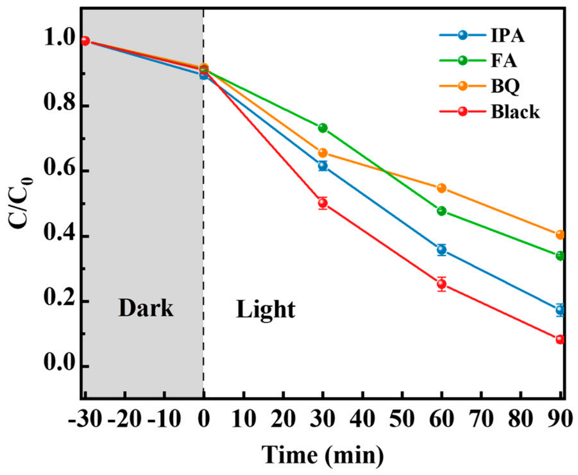

Figure S12. Photocatalytic degradation of MB by 6-*g*-C_3N_4/BiOBr in the presence of different scavengers: IPA for quenching ·OH, BQ for quenching $·O_2^-$, and FA for quenching h^+.

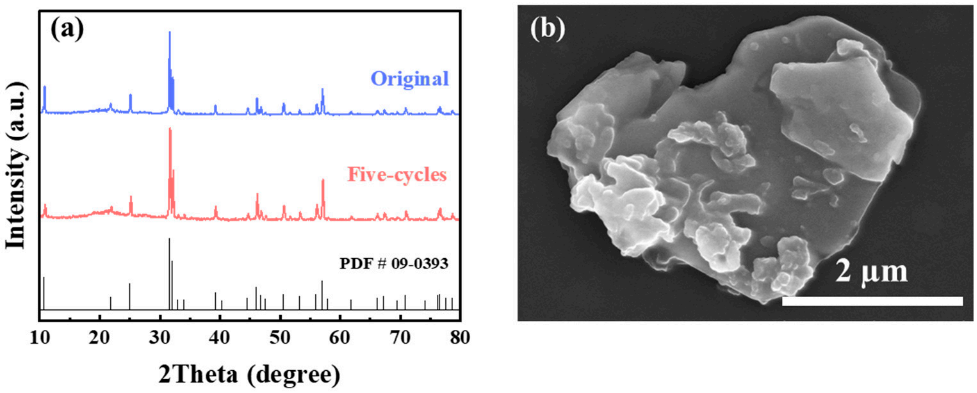

Figure S13. (a) XRD and (b) SEM of 6-*g*-C_3N_4/BiOBr catalyst before and after cycling
